# Causal relationships between antibody-induced immune responses and sepsis: Evidence from genetic studies

**DOI:** 10.1097/MD.0000000000047458

**Published:** 2026-02-13

**Authors:** Liqun Li, Lijian Liu, Jing Yan, Jinjing Tan, Sheng Xie

**Affiliations:** a The First Affiliated Hospital of Guangxi University of Chinese Medicine, Nanning, Guangxi, China; b Graduate School of Guangxi University of Chinese Medicine, Nanning, Guangxi, China.

**Keywords:** antibody-mediated immune responses, inflammatory cytokines, mediators, sepsis, 2-sample bidirectional Mendelian randomization

## Abstract

The causal relationships between antibody-induced immune responses and the occurrence and mortality of sepsis remain controversial. The 2-sample Mendelian randomization (MR) approach was utilized to reveal the causal associations, along with the potential mediation effects of inflammatory cytokines. The causal associations were analyzed by a 2-sample bidirectional MR analysis, primarily using the inverse variance weighted method. MR-Egger regression, weighted mode, weighted median, and simple mode were conducted as supplementary analyses. Additionally, we performed a 2-step MR to investigate the potential mediation effects of 91 inflammatory cytokines. Cochran *Q* test was conducted to assess statistical heterogeneity. Potential horizontal pleiotropy was identified with MR-Egger regression intercept test and MR-pleiotropy residual sum and outlier global test. Leave-one-out sensitivity analysis was employed to evaluate the influence of an individual single nucleotide polymorphism on the estimates. The outcomes revealed positive associations between genetically predicted *Helicobacter pylori* urea antibody levels (odds ratios [ORs] = 1.070, 95% confidence interval [CI]: 1.009–1.134, *P* = .024), anti-herpes simplex virus type 1 immunoglobulin G seropositivity [OR = 1.071, 95% CI: 1.012–1.134, *P* = .018], and the risk of sepsis; cytomegalovirus phosphoprotein 52 antibody levels showed significant negative correlation with 28-day mortality in sepsis [OR = 0.830, 95% CI: 0.690–0.999, *P* = .048]. Surprisingly, the mediation analysis suggested that the 91 inflammatory cytokines did not mediate these associations. *H pylori* urea antibody and anti-herpes simplex virus type 1 immunoglobulin G seropositivity are pathogenic factors for sepsis, while cytomegalovirus phosphoprotein 52 antibody levels may protect against 28-day mortality in sepsis. Inflammatory cytokines may not mediate these relationships. These findings could contribute to the precise management of sepsis.

## 1. Introduction

Sepsis is a life-threatening clinical syndrome characterized by organ dysfunction resulting from dysregulated host immune and inflammatory responses to infection.^[1]^ It can be triggered by various infectious diseases, including pneumonia, peritonitis, and urinary tract infections, making it a prevalent global health concern. Epidemiological data indicate a substantial disease burden, with a global incidence of 205 cases per 100,000 person-years. In 2017 alone, approximately 48.9 million sepsis cases were reported worldwide, alongside 11 million sepsis-related deaths, accounting for 19.7% of global mortality.^[2]^ Although mortality rates have seen a recent decline, sepsis remains a leading cause of death, with a reported 30-day mortality reaching 17.4%, increasing to 26% to 32.6% in cases of severe sepsis, particularly in low- and middle-income countries.^[3–5]^ Furthermore, studies have suggested that up to 41.7% to 41.9% of sepsis patients die in hospitals.^[6,7]^ In intensive care unit settings, mortality associated with severe sepsis or septic shock remains as high as 34.3% to 40.4%,^[8]^ underscoring the urgent need to better understand its pathogenesis and improve clinical outcomes.

Although bacterial and fungal infections are primary causes of sepsis, non-bacterial pathogens contribute significantly, accounting for up to 42% of cases.^[9]^ Viral infections, in particular, play a crucial role. Herpes simplex viruses (HSV) are major etiological agents in neonatal sepsis.^[9,10]^ Among adult sepsis patients, 33.3% are positive for HSV type 1 (HSV-1), 42.1% for cytomegalovirus (CMV), 60.6% for Epstein–Barr virus (EBV), and 98.4% for human herpesvirus 6 (HHV-6).^[11]^ CMV reactivation influences bacterial processes and correlates with prolonged hospitalization and increased mortality.^[12–15]^ Similarly, EBV reactivation is associated with extended intensive care unit stays and worsened organ dysfunction.^[16]^ A cohort study reported that CMV and EBV seropositivity increase the risk of sepsis mortality by 3.01-fold and 6.10-fold, respectively.^[11]^ Nevertheless, some studies report no significant effect of CMV reactivation on overall sepsis mortality,^[17]^ indicating ongoing controversy regarding the role of viral infections. Other non-bacterial pathogens, such as *Chlamydia trachomatis* and *Helicobacter pylori*, have also been implicated in sepsis pathogenesis and outcomes, though evidence remains limited due to confounding factors.^[18,19]^ Identifying causative pathogens is essential for predicting disease progression and refining treatment strategies.

The core mechanism of sepsis involves maladaptive host responses to infection, marked by inflammatory and immune dysregulations.^[20,21]^ Systemic immune dysfunction underlies severe sepsis and septic shock, wherein microbial or self-antigens trigger the humoral immune activation and excessive inflammation.^[22]^ Antibodies, produced in response to pathogens, modulate immune activity upon binding to antigens or cellular targets.^[23,24]^ Seropositivity can indicate prior pathogen exposure and provide insights into the pathophysiology of sepsis.^[25]^ However, antibody-mediated immunity may play dual roles: certain autoantibodies can cause immune-mediated damage,^[26]^ while in other contexts, immunosuppression is observed following CMV reactivation, accompanied by elevated levels of cytokines such as interleukin (IL)-6 and IL-10.^[27]^ Reduced anti-CMV immunoglobulin G and anti-CMV glycoprotein B immunoglobulin G titers have also been reported in sepsis patients.^[28]^ These findings suggest that antibody responses are involved in the pathology of sepsis, though observational studies are limited by small sample sizes, confounding factors, and individual variability. Thus, the causal relationship between antibody-mediated immunity and sepsis remains unclear.

Mendelian randomization (MR) uses genetic variants associated with exposure traits as the instrumental variables (IVs) to infer causality, minimizing confounding, and reverse causation that are typical of observational studies.^[29–31]^ Bidirectional two-sample MR (TSMR) further strengthens causal inference. In this study, we applied TSMR to investigate whether antibody-mediated immune responses causally influence sepsis risk and 28-day mortality, and whether inflammatory cytokines mediate these relationships. To our knowledge, this represents the 1st comprehensive MR analysis to address these questions, aimed at informing future sepsis management strategies.

## 2. Materials and methods

### 2.1. Study design and assumption

**Figure 1.**
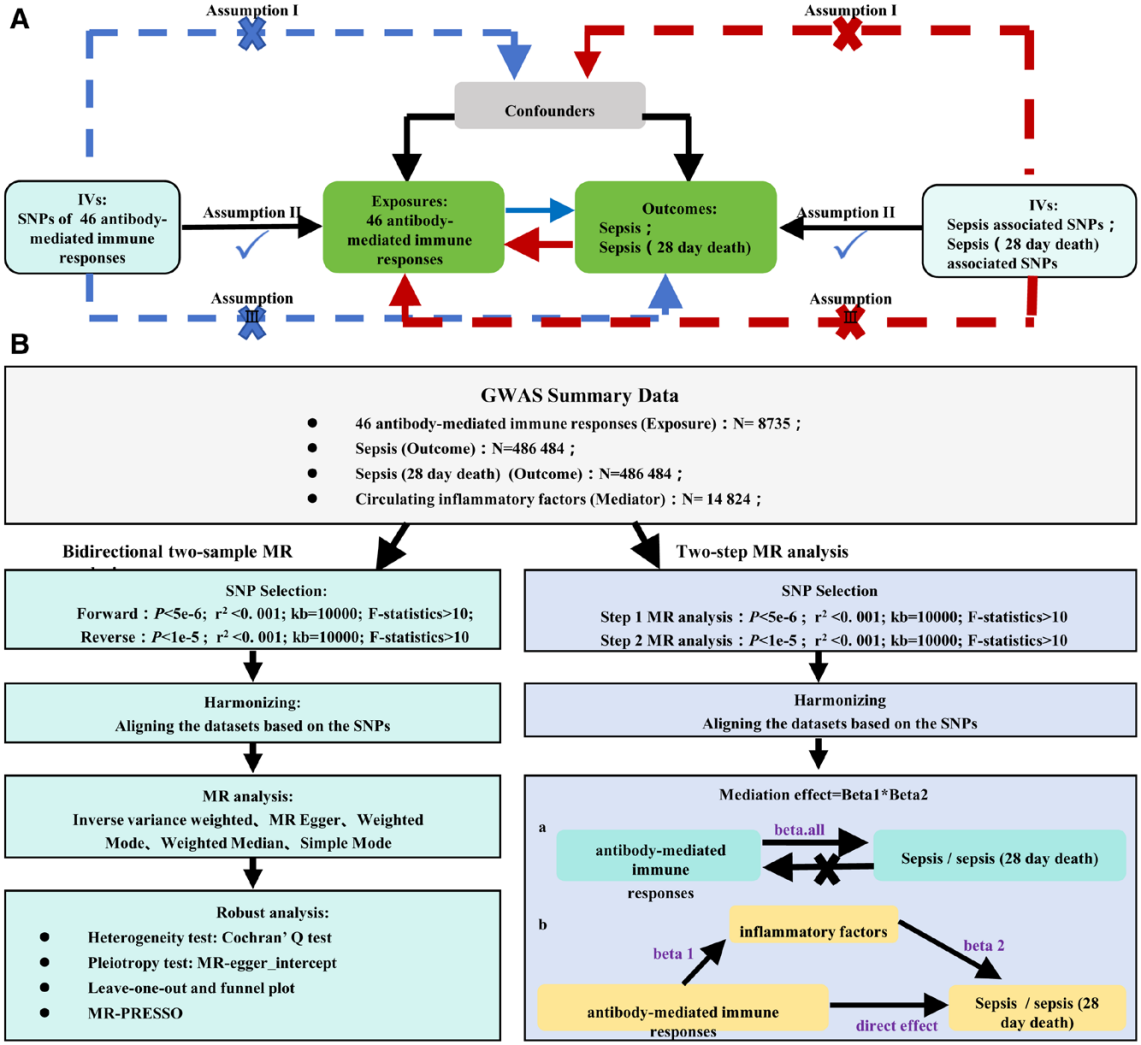
(A) Schematic diagram of Mendelian randomization (MR) principles. (B) Flowchart of 2-sample MR analysis and mediation analysis.

This study employed TSMR analysis utilizing 46 antibody-mediated immune response-related, sepsis and sepsis-28-day death-associated single nucleotide polymorphisms (SNPs) as IVs. First, we assessed the causal relationship between the 46 antibody-mediated immune responses, sepsis and sepsis-related 28-day death through TSMR analysis. Subsequently, a 2-step MR analysis, mediated by 91 inflammatory cytokines, was performed to reveal the exact role of these cytokines in the aforementioned causal relationships. Our study was based on 3 assumptions of MR^[32]^: IVs have no correlations with confounding factors; IVs are closely associated with the exposure; IVs exclusively influence the outcome via the exposure. All data used in this research were publicly available, therefore obviating the need for additional ethical approval. In addition, the data were obtained from publicly available data from previous publications or collaborative datasets and do not require registration with a clinical trial registry. We adhered to the reporting standards outlined in the Strengthening the Reporting of Observational Studies in Epidemiology-MR guidelines for MR studies.^[33]^ The study flow diagram is illustrated in Figure 1.

### 2.2. GWAS data sources

The 2 datasets involved in this MR analysis were sourced from the publicly available genome-wide association study (GWAS) databases. The GWAS data for the 46 antibody-mediated immune responses were derived from a published cohort GWAS study, which included 8735 samples with 10,000 serological measurements for infectious diseases and whole-genome genotyping.^[25]^ Data for 91 circulating inflammatory cytokines were derived from 11 GWAS cohorts, with a total of 14,824 participants.^[34]^ Additionally, the GWAS summary data for sepsis and sepsis-28-day death were retrieved from the IEU Open GWAS project (https://gwas.mrcieu.ac.uk/), with the corresponding GWAS IDs ieu-b-4980 and ieu-b-5086. The definitions of sepsis and sepsis-related 28-day death were based on hospital discharge records (consistent with ICD codes and Sepsis-3 criteria) and all-cause mortality records from respective national registry, respectively. These datasets included a total of 486,484 participants. In this study, MR analysis was conducted exclusively in European participants, thereby mitigating confounding factors arising from racial and regional disparities.

### 2.3. Genetic instruments

We set a genome-wide significance threshold of *P* < 5e-6 to identify highly relevant SNPs for the condition, where antibody-mediated immune responses were considered the exposure. For sepsis as the exposure, we set a genome-wide significance threshold of *P* < 1e-5 to select SNPs. Additionally, a significance threshold of *P* < 1e-5 was set to screen for significant SNPs when sepsis-related 28-day mortality was the exposure. Significant SNPs (*P* < 1e-5) were obtained from the genome-wide analysis of 91 circulating inflammatory cytokines. These screening conditions ensured a strong correlation between the selected IVs and the exposures. To ensure the independent selection of SNPs and minimize the impact of linkage disequilibrium, we set thresholds of *R* = 0.001 and 10,000 kb for genetic distance. Furthermore, we set the *F*-statistics threshold at *F* > 10 to mitigate bias caused by weak IVs. The characteristics of these SNPs are listed in Tables S1 to S4, Supplemental Digital Content, https://links.lww.com/MD/R273.

### 2.4. Bidirectional MR analysis

TSMR analysis was conducted to assess the causal associations between antibody-mediated immune responses and sepsis, as well as sepsis-related 28-day mortality. First, in forward MR analysis, genetic variants associated with antibody-mediated immune responses were used as the exposure to examine the causal effects of genetically predicted antibody-mediated immune responses on sepsis and sepsis-related 28-day mortality. In reverse MR analysis, genetic variants related to sepsis and sepsis-related 28-day mortality were selected as the exposure to evaluate the influence of these variants on antibody-mediated immune responses. The inverse variance weighted (IVW),^[35,36]^ MR-Egger regression (MR-Egger),^[37]^ weighted mode,^[38]^ weighted median,^[39]^ and simple mode methods were employed for MR analysis. Since IVW is considered the most effective approach for assessing MR results,^[35,36]^ our statistical analyses primarily relied on IVW statistics to determine the significance of the findings. All MR analyses were performed using the TwoSampleMR and the MR-PRESSO packages in R software (Version 4.2.3; R Foundation for Statistical Computing, Vienna, Austria).

### 2.5. Sensitivity analysis

Multiple sensitivity analyses were performed in this study, including a heterogeneity test, a horizontal pleiotropy test, and a leave-one-out (LOO) sensitivity analysis. First, Cochran *Q* test was performed to assess heterogeneity, where *P* > .05 indicated no evidence of significant heterogeneity.^[35,40,41]^ Second, we used MR-PRESSO method to detect and exclude potential outliers, thereby calibrating horizontal pleiotropy.^[42]^ Additionally, the MR-Egger intercept test was applied to assess directional pleiotropy. In cases of discordant results among these pleiotropy tests, priority was given to the MR-PRESSO-corrected estimates, as this method directly identifies and removes outliers. Finally, a LOO test was performed to evaluate the stability of effect sizes by excluding each SNP individually.

### 2.6. Mediation analysis with TSMR

Mediation analysis was performed via TSMR to explore the potential crucial mediating effect of 91 circulating inflammatory cytokines on the causal relationship between antibody-mediated immune responses and sepsis, as well as sepsis-related 28-day mortality. First, MR analysis was performed to examine the relationship between antibody-mediated immune responses and 91 circulating inflammatory cytokines, aiming to identify the antibody immune response phenotypes with significant effect estimates but no pleiotropy, and yielding Beta 1 values. Subsequently, MR analysis was performed to examine inflammatory cytokines that were significantly associated with sepsis and sepsis-related 28-day mortality and exhibited no pleiotropy, generating Beta 2 values. Finally, Beta_all was obtained via MR analysis examining the relationship between antibody-mediated immune responses and both sepsis and sepsis-related 28-day mortality. The mediation effect was calculated with the formula: Beta 1 × Beta 2. The mediation analysis process is illustrated in Figure 1.

## 3. Results

### 3.1. Genetic instruments selection

In the forward MR analysis, a series of screenings steps were applied to SNPs associated with 46 antibody-mediated immune responses, following the aforementioned screening conditions, and the results are detailed in Table S1, Supplemental Digital Content, https://links.lww.com/MD/R273. For each antibody-mediated immune response, the number of SNPs ranged from 5 to 59, and the *F*-statistics ranged from 20.776 and 343.063. This indicates a strong relationship between the SNPs and the corresponding antibody-mediated immune responses (*P* < 5e-6). For the reverse MR analysis, 21 SNPs strongly associated with sepsis (*P* < 1e-5) and 27 SNPs strongly associated with sepsis-related 28-day mortality (*P* < 1e-5) were selected using the same approach from summary data of the European population (all *F*-statistics > 10) (Tables S2 and S3, Supplemental Digital Content, https://links.lww.com/MD/R273). In the TSMR-based mediation analysis of 91 inflammatory cytokines, a threshold of *P* < 1e-5 was set to select SNPs significantly associated with the inflammatory cytokines, with the number of SNPs ranging from 5 to 44 (all *F*-statistics > 10) (Table S4, Supplemental Digital Content, https://links.lww.com/MD/R273). In conclusion, all SNPs involved in this study exhibited a strong correlation with their respective exposures and were free of linkage disequilibrium or pleiotropy effects, confirming them as robust IVs.

### 3.2. Forward MR analysis

**Figure 2.**
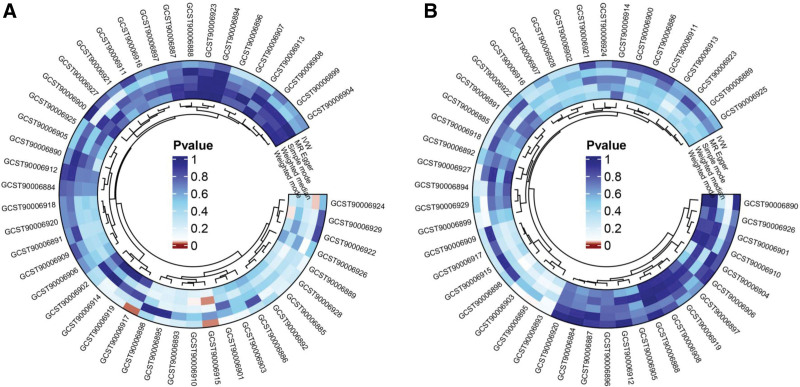
(A) Circo plot showing causal correlations between 46 antibody-mediated immune responses and sepsis (forward MR analysis). (B) Circo plot showing causal associations between 46 antibody-mediated immune responses and sepsis-related 28-day mortality (forward MR analysis). MR = Mendelian randomization.

**Figure 3.**
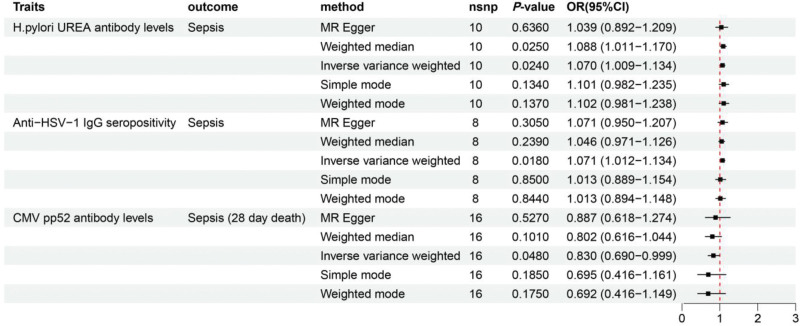
Forest plot showing significant causal associations between specific antibody-mediated immune responses and sepsis, as well as sepsis-related 28-day mortality (forward MR analysis). MR = Mendelian randomization.

SNPs associated with 46 antibody-mediated immune responses were used to analyze the causal relationships between antibody-mediated immune responses and sepsis. Table S5, Supplemental Digital Content, https://links.lww.com/MD/R273 presents the positive MR analyses results via various methods. The primary IVW results revealed a positive association between 2 antibody-mediated immune responses and sepsis, whereas genetic variants associated with the remaining 44 antibody-mediated immune responses showed no significant association with sepsis (Fig. 2A and Figure S1, Supplemental Digital Content, https://links.lww.com/MD/R274). Specifically, *H pylori* urea antibody levels (odds ratios [ORs] = 1.070, 95% confidence interval [CI]: 1.009–1.134, *P* = .024) and anti-herpes simplex virus type 1 immunoglobulin G (anti-HSV-1 IgG) seropositivity [OR = 1.071, 95% CI: 1.012–1.134, *P* = .018] were significantly associated with an increased risk of sepsis (Fig. 3). The reliability of these causal relationships was also confirmed using MR-PRESSO (Table S6, Supplemental Digital Content, https://links.lww.com/MD/R273).

Subsequently, a forward MR analysis was performed to examine the correlation between 46 antibody-mediated immune responses and sepsis-related 28-day mortality. The results of different MR methods are demonstrated in Table S7, Supplemental Digital Content, https://links.lww.com/MD/R273. According to the IVW results, we identified only 1 antibody-mediated immune response that showed a negative association with sepsis-related 28-day mortality. In contrast, no statistically significant causal relationships were detected between the remaining 45 antibody-mediated immune responses and the risk of sepsis-related 28-day mortality (Fig. 2B and Figure S2, Supplemental Digital Content, https://links.lww.com/MD/R274). Specifically, CMV phosphoprotein 52 (CMV pp52) antibody levels showed a significant negative association with sepsis-related 28-day mortality [OR = 0.830, 95% CI: 0.690–0.999, *P* = .048]. This suggests that genetic susceptibility to this antibody-mediated immune response may reduce the risk of sepsis-related 28-day mortality (Fig. 3).

### 3.3. Reverse MR analysis

**Figure 4.**
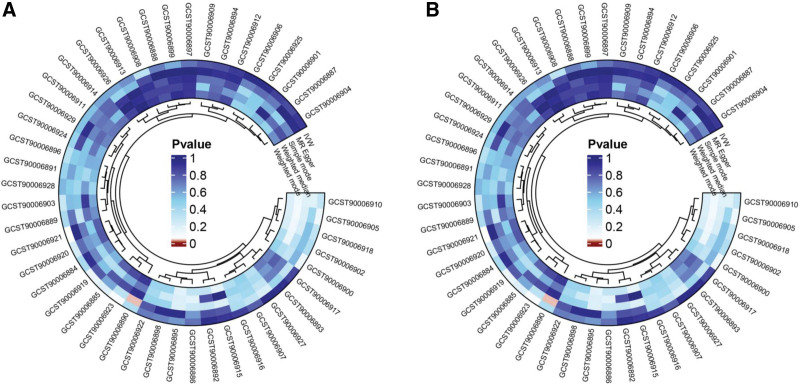
(A) Circos plot showing causal associations between 46 antibody-mediated immune responses and sepsis (reverse MR analysis). (B) Circos plot showing causal associations between 46 antibody-mediated immune responses and sepsis-related 28-day mortality (reverse MR analysis). MR = Mendelian randomization.

Reverse MR analysis was performed to explore the causal relationships between genetic susceptibility to sepsis and antibody-mediated immune responses. The results of various MR methods are presented in Table S8, Supplemental Digital Content, https://links.lww.com/MD/R273. IVW statistics indicated no significant effect of genetic susceptibility to sepsis on any of the 46 antibody-mediated immune responses (*P* > .05) (Fig. 4A and Figure S3, Supplemental Digital Content, https://links.lww.com/MD/R274). Furthermore, the global test results from MR-PRESSO further supported this conclusion (Table S9, Supplemental Digital Content, https://links.lww.com/MD/R273).

We further evaluated the causal relationships between genetic susceptibility to sepsis-related 28-day mortality and antibody-mediated immune responses. The results of different MR methods are presented in Table S10, Supplemental Digital Content, https://links.lww.com/MD/R273. Genetic susceptibility to sepsis-related 28-day mortality was found to have a significant effect on 2 specific antibody-mediated immune responses; however, no statistically significant causal relationships were detected between this susceptibility and the other 44 antibody-mediated immune responses. These immune responses included levels of CMV pp52 antibodies [OR = 1.023, 95% CI: 0.973–1.075, *P* = .374] and CMV pp150 antibodies [OR = 1.005, 95% CI: 0.953–1.061, *P* = .845] (Fig. 4B and Figure S4, Supplemental Digital Content, https://links.lww.com/MD/R274).

**Figure 5.**
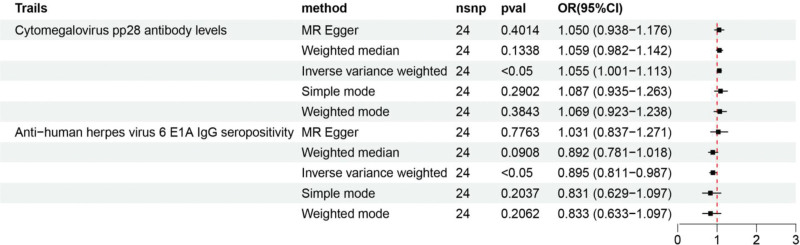
Forest plot showing causal associations between 46 antibody-mediated immune responses and sepsis-related 28-day mortality (reverse MR analysis). MR = Mendelian randomization.

Specifically, a significant positive association was detected between sepsis-related 28-day mortality and CMV pp28 antibody levels [OR = 1.055, 95% CI: 1.001–1.113, *P* = .045], whereas a strong negative association was detected between this mortality and anti-human herpes virus (HHV) 6 E1A IgG seropositivity [OR = 0.895, 95% CI: 0.811–0.987, *P* = .026] (Fig. 5).

### 3.4. Sensitivity analysis

Different methods were applied to assess potential heterogeneity and horizontal pleiotropy in the MR results. The results of sensitivity analyses for MR analyses are respectively displayed in Table S11 and S12, Supplemental Digital Content, https://links.lww.com/MD/R273. Although heterogeneity was detected in several statistics analyses via Cochran *Q* test, the results remained consistent after adjustment using a random-effects model. The MR-Egger intercept test found horizontal pleiotropy in a few causal estimates (*P* < .05), after removing outlier SNPs and adjusting the analyses via the MR-PRESSO global test, the pleiotropy was no longer detected (*P* > .05). Therefore, the consistency between the adjusted results and the original findings confirms the robustness of the MR analysis employed in this study.

**Figure 6.**
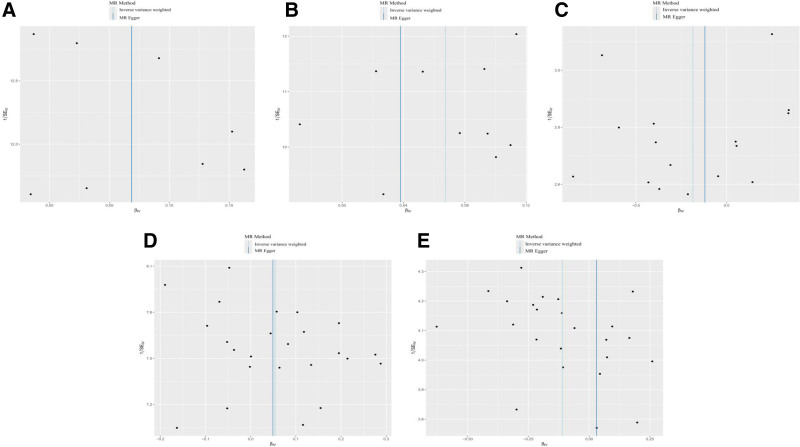
Funnel plots. (A) Funnel plot of *H pylori* urea antibody levels and sepsis. (B) Funnel plot of anti-HSV-1 IgG seropositivity and sepsis. (C) Funnel plot of CMV pp52 antibody levels and sepsis-related 28-day mortality (non-sepsis patients). (D) Funnel plot of sepsis-related 28-day mortality and CMV pp28 antibody levels. (E) Funnel plot of sepsis-related 28-day mortality and anti-HHV-6 E1A IgG seropositivity. Anti-HSV-1 IgG = anti-herpes simplex virus type 1 immunoglobulin G, CMV pp52 = cytomegalovirus phosphoprotein 52, HHV = anti-human herpes virus, HHV-6 = human herpesvirus 6, HSV = herpes simplex viruses, HSV-1 = herpes simplex virus type 1.

**Figure 7.**
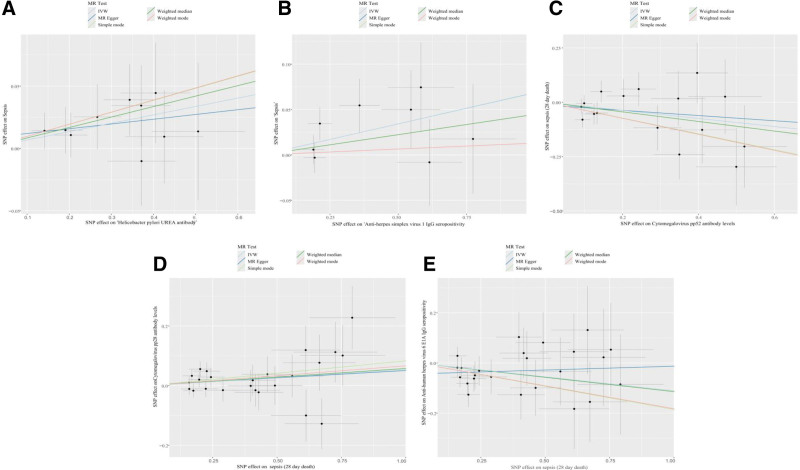
Scatter plots. (A) Scatter plot of *H pylori* urea antibody levels and sepsis. (B) Scatter plot anti-HSV-1 IgG seropositivity and sepsis. (C) Scatter plot of CMV pp52 antibody levels and sepsis-related 28-day mortality (non-sepsis patients). (D) Scatter plot of sepsis-related 28-day mortality and CMV pp28 antibody levels. (E) Scatter plot of sepsis-related 28-day mortality and anti-HHV-6 E1A IgG seropositivity. Anti-HSV-1 IgG = anti-herpes simplex virus type 1 immunoglobulin G, CMV pp52 = cytomegalovirus phosphoprotein 52, HHV = anti-human herpes virus, HHV-6 = human herpesvirus 6, HSV = herpes simplex viruses, HSV-1 = herpes simplex virus type 1.

**Figure 8.**
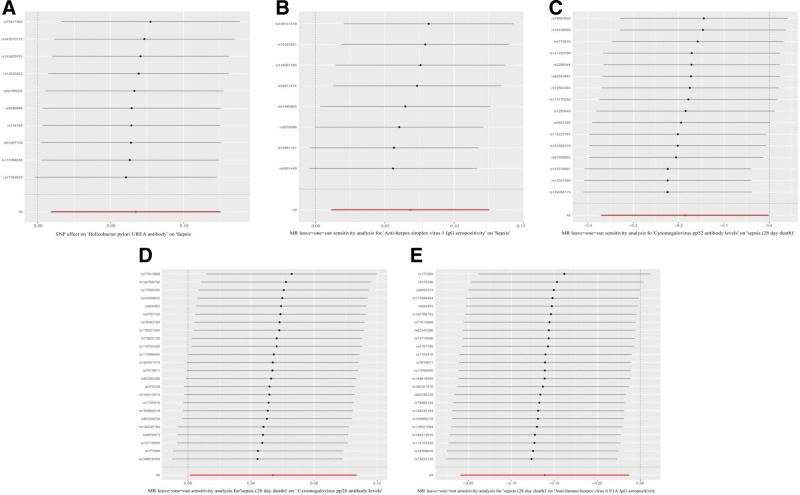
Leave-one-out (LOO) sensitivity analyses. (A) LOO sensitivity analysis of *H pylori* urea antibody levels and sepsis. (B) LOO sensitivity analysis of anti-HSV-1 IgG seropositivity and sepsis. (C) LOO sensitivity analysis of CMV pp52 antibody levels and sepsis-related 28-day mortality (non-sepsis patients). (D) LOO sensitivity analysis of sepsis-related 28-day mortality and CMV pp28 antibody levels. (E) LOO sensitivity analysis ofsepsis-related 28-day mortality and anti-HHV-6 E1A IgG seropositivity. CMV pp52 = cytomegalovirus phosphoprotein 52, HHV-6 = human herpesvirus 6, HSV = herpes simplex viruses, HSV-1 = herpes simplex virus type 1.

Notably, the funnel plots did not exhibit significant heterogeneity in the positive results (Fig. 6). In the forward MR analysis, scatter plots demonstrated a positive association between *H pylori* urea antibody and anti-HSV-1 IgG seropositivity, and sepsis risk. In contrast, CMV pp52 antibody levels exhibited a significant negative association with sepsis-related 28-day mortality. In the reverse MR analysis, scatter plots showed a significant positive relationship between sepsis-related 28-day mortality and CMV pp28 antibody levels. Meanwhile, a strong negative association was observed between this mortality and anti-HHV-6 E1A IgG seropositivity (Fig. 7). The results of the LOO sensitivity analysis showed that the MR results were not driven by any individual SNP (Fig. 8). In summary, the consistency of sensitivity analysis results supports the validity of the causal inferences from the primary analysis, and non-significant forward MR results are less likely to be confounded by reverse causality.

### 3.5. Mediation analysis

The results of bidirectional TSMR analysis confirmed that *H pylori* urea antibody levels and anti-HSV-1 IgG seropositivity significantly increased the risk of sepsis, whereas CMV pp52 antibody levels significantly reduced the risk of sepsis-related 28-day mortality. However, whether these causal relationships are mediated by specific factors remains unclear. Given that immune-inflammatory dysregulation acts as the core pathological mechanism of sepsis, a 2-step MR analysis utilizing 91 circulating inflammatory mediators was performed in this study to assess whether they serve as mediators of these causal relationships.

First, MR analysis was performed to examine the relationship between the aforementioned antibody-mediated immune responses and 91 circulating inflammatory cytokines. The statistical outcomes from different MR methods are presented in Table S13, Supplemental Digital Content, https://links.lww.com/MD/R273. Preliminary IVW results revealed that *H pylori* urea antibody levels were positively associated with levels of T lymphocyte (T-cell) surface glycoprotein CD6 isoform (CD6) (*P* = .015), IL-20 receptor subunit alpha (*P* = .047), IL-2 receptor subunit beta (*P* = .008), and thymic stromal lymphopoietin (*P* = .005), but negatively associated with IL-33 (*P* = .040), oncostatin M (*P* = .018), and transforming growth factor-alpha (*P* = .047). Concurrently, anti-HSV-1 IgG seropositivity showed a positive correlation with leukemia inhibitory factor receptor (*P* = .046) and a negative correlation with IL-20 receptor subunit alpha (*P* = .016). CMV pp52 antibody levels were positively associated with IL-17A (*P* = .014) and IL-4 (*P* = .022). However, these inflammatory cytokines did not exhibit significant causal relationships with sepsis or sepsis-related 28-day mortality (Table S14, Supplemental Digital Content, https://links.lww.com/MD/R273). Sensitivity analysis did not reveal significant heterogeneity or horizontal pleiotropy either (Table S15, Supplemental Digital Content, https://links.lww.com/MD/R273).

Second, we performed an MR analysis of 91 inflammatory factors to examine the causal relationship between sepsis and sepsis-related 28-day mortality. The statistical results for different MR methods are demonstrated in Table S14, Supplemental Digital Content, https://links.lww.com/MD/R273. IVW results showed a positive association of IL-5 (*P* = .023) and vascular endothelial growth factor A (*P* = .027), and sepsis, whereas tumor necrosis factor beta (*P* = .027) showed a negative association (Table S14, Supplemental Digital Content, https://links.lww.com/MD/R273). CD6 (*P* = .008), IL-12 subunit beta (*P* = .035), IL-18 (*P* = .035), monocyte chemoattractant protein-1 (*P* = .033), matrix metalloproteinase-10 (*P* = .038), and transforming growth factor-alpha (*P* = .009) may decrease the risk of sepsis-related 28-day mortality. The results of sensitivity analyses suggest that these causal relationships are robust (Table S16, Supplemental Digital Content, https://links.lww.com/MD/R273). However, these inflammatory cytokines were not influenced by *H pylori* urea antibody levels, anti-HSV-1 IgG seropositivity, or CMV pp52 antibody levels (Table S13, Supplemental Digital Content, https://links.lww.com/MD/R273).

In conclusion, there was no evidence that inflammatory factors mediated these significant causal relationships.

## 4. Discussion

To our knowledge, this study represents the largest investigation using TSMR to explore the potential causal relationships between 46 antibody-mediated immune responses and both sepsis and sepsis-related 28-day mortality. Additionally, it is also the largest 2-step MR analysis of 91 circulating inflammatory cytokines to explore their mediating effects. According to our substantial findings, elevated levels of *H pylori* urea antibody and anti-HSV-1 IgG seropositivity were associated with an increased risk of sepsis, while elevated CMV pp52 antibody levels were associated with a reduced risk of sepsis-related 28-day mortality. Reverse MR analysis revealed a significant positive association between sepsis-related 28-day mortality and CMV pp28 antibody levels, as well as a significant negative association with anti-HHV-6 E1A IgG seropositivity. Importantly, sensitivity analyses showed no significant heterogeneity or horizontal pleiotropy, confirming the robustness of our results. Furthermore, mediation analysis suggested that these inflammatory cytokines may not serve as mediators in the aforementioned causal relationships. In conclusion, our study provides valuable insights into the role of antibody-mediated immune responses in the management of sepsis. The modest ORs observed in our study (e.g., ~1.07 for sepsis risk) are a common feature of MR analyses, as genetic instruments typically explain a limited fraction of the phenotypic variance. These estimates represent the lifelong effect of a genetically predisposed antibody-mediated immune response. While their magnitude may not indicate immediate clinical utility for individual risk prediction, they hold significant value by unveiling causal directions and prioritizing specific antibody-mediated immune responses (e.g., those against *H pylori* urea and HSV-1) for future mechanistic and preventive research.

*H pylori* is a gram-negative bacterium known for its global prevalence that causes infectious diseases. Studies have reported an infection rate ranging from 18.9% to 87.7%, affecting approximately 4.4 billion people worldwide in 2015.^[43]^ *H pylori* infection can be associated with pathogens linked to various human diseases.^[44]^ Previous studies have suggested a potential relationship between *H pylori* and sepsis, but the strength of the evidence is limited.^[18]^ Moreover, there have not yet been any large-scale prospective clinical studies demonstrating a causal association between *H pylori* infection and sepsis. Our TSMR analysis provides genetic evidence supporting a significant association between *H pylori* urea antibody levels and an increased risk of sepsis, suggesting a potential causal relationship. *H pylori* infection is characterized by inflammatory infiltration of tissues. Research indicates that *H pylori* infection activates the NOD-like receptor pyrin domain-containing protein 3 inflammasome,^[45–47]^ leading to the production of the IL-1 beta, IL-17A, IL-1, IL-18, and IL-10.^[45,46,48,49]^ Evidence also suggests a crucial role of the NOD-like receptor pyrin domain-containing protein 3 inflammasome in the onset and progression of sepsis,^[50,51]^ while IL-17A and the IL-1 family (e.g. IL-1α, IL-1β, IL-33) may promote sepsis development.^[52,53]^ These findings suggest that inflammatory factors may act as mediators in the causal association between *H pylori* infection and sepsis. However, our 2-step MR analysis results do not support this hypothesis; specifically, the mediation analysis found no evidence that inflammatory cytokines mediate the increased risk of sepsis associated with *H pylori* urea antibody levels. Other mediating factors may be involved in this causal association, such as gut microbiota and cellular immunity. Scholars have proposed that pathogenic factors of *H pylori* affect gut microbiota, thereby contributing to the development of several extra-gastric diseases.^[54–56]^ In sepsis, alterations in gut microbiota lead to increased intestinal permeability and a dysregulated immune response to sepsis, thereby increasing death.^[57]^ A MR analysis has indicated a causal association between gut microbiota and both sepsis and sepsis-related 28-day mortality.^[58]^ Furthermore, *H pylori* may inhibit T cells function by releasing outer membrane vesicles.^[59]^ T-cell-mediated immune suppression is a critical pathological mechanism in sepsis.^[60]^ The above evidence suggests that gut microbiota and cellular immunity may mediate the association between *H pylori* urea antibody levels and sepsis risk. Future research will further explore the mediating roles of gut microbiota and cellular immunity in this causal relationship using 2-step MR analysis. Large-scale prospective clinical studies should be conducted to further confirm the association between *H pylori* urea antibody levels and an increased risk of sepsis. This would contribute to the prevention and treatment of sepsis, thereby reducing both its incidence and mortality rates.

The MR analysis has confirmed that elevated anti-HSV-1 IgG levels are associated with an increased risk of sepsis. HSV is a globally prevalent human pathogen. Once infected, it establishes latency in neurons and may cause diseases such as herpes and encephalitis during primary infection and reactivation.^[61]^ HSV-1 is the main subtype of HSV. Epidemiological studies have estimated that in 2016, 3.752 billion people were infected with HSV-1, with a global prevalence of 66.6% in the 0 to 49 age group, even reaching as high as 99.2% in some regions.^[62,63]^ Clinically, IgG antibody levels are often considered indicative of an individual’s immune response to specific pathogens. In some cases, generated IgG antibodies also serve as endogenous antigens and thereby inducing anti-IgG production, further affecting the immune system. Substantial evidence has suggested that autoantibodies can promote the development of autoimmune diseases and, therefore, be utilized utilized as predictors.^[64,65]^ Anti-HSV-1 IgG antibodies are produced 21 to 28 days after exposure and may persist throughout an individual’s lifetime.^[66]^ Studies have shown that up to 96.7% of healthy individuals test positive for anti-HSV-1 IgG.^[67]^ Anti-HSV-1 IgG levels are associated with various diseases, such as duodenal ulcer perforation.^[68]^ An previous MR analysis indicated an association between HSV-1 susceptibility and an increased risk of sepsis.^[69]^ That study focuses on the correlation between HSV-1 infection and sepsis risk, while our MR analysis focused on the causal relationship between HSV-1-related antibodies and sepsis risk, highlighting their differences. To our knowledge, no direct evidence linking anti-HSV-1 IgG positivity to sepsis risk has been identified to date. Our study provides genetic evidence supporting a significant association between positive anti-HSV-1 IgG and an increased risk of sepsis. Further exploration of the mechanisms underlying this correlation remains necessary in future studies, but the involvement of inflammatory mechanisms is excluded by our mediation analysis.

Collectively, our null mediation finding strategically redirects the investigative focus away from inflammatory cytokines and toward other biological pathways. The mechanisms discussed herein, particularly the modulation of gut microbiota^[57,58]^ and T-cell-mediated immunity,^[59,60]^ emerge as the most compelling candidates for explaining the observed causal relationships. Future research should prioritize elucidating these specific alternative pathways.

CMV is the most common opportunistic virus in immunocompromised hosts and a prevalent viral pathogen in patients with sepsis. Active CMV infection has been detected in 13.69% to 24.2% of sepsis patients, and CMV antibodies have been detected in 42.1% of children and adolescents with sepsis.^[11,15,70]^ Studies have indicated that CMV infection is associated with poor prognosis in sepsis, and CMV reactivation can exacerbate organ dysfunction, significantly increasing 28- and 90-day mortality rates among sepsis patients.^[15,70,71]^ However, our forward MR analysis yielded contradictory results, suggesting that elevated CMV pp52 antibody levels were associated with a reduced28-day mortality risk in sepsis patients, with no evidence of reverse causation. Sensitivity analyses confirmed the robustness of our MR findings. These contradictory results are likely influenced by confounding factors such as age, timing of infection, and concurrent infections, highlighting the low evidence strength of previous observational clinical studies. Based on Mendel laws of inheritance, our MR analysis aimed to mitigate common confounding factors and address reverse causation, thereby providing more reliable evidence for our conclusions. It is important to note that the protective association between CMV pp52 antibody levels and 28-day sepsis mortality (OR = 0.830, 95% CI: 0.690–0.999, *P* = .048) must be interpreted with caution. The CI is wide and includes the null value at its upper bound, indicating statistical uncertainty. Therefore, this finding should be considered hypothesis-generating and requires validation in larger independent patient cohorts. In terms of mechanisms, however, our mediation analysis did not support the involvement of inflammatory cytokines. Host immune dysregulation is known as the core mechanism leading to organ dysfunction and mortality in sepsis patients.^[72]^ Lymphocytes are the primary cells mediating host immune responses. Research has indicated that an augmented humoral response to CMV pp52 is associated with CMV activity, and a positive association has been observed between CMV pp52-guided IgG levels and lymphocyte counts in patients with systemic lupus erythematosus.^[73]^ Additionally, CD8+ and CD28 + T-cell counts are negatively correlated with CMV deoxyribonucleic acid negativity conversion.^[70]^ Therefore, it can be inferred that CMV pp52-mediated immune responses may increase lymphocyte counts. Studies have reported a significant decrease in absolute lymphocyte counts in sepsis patients and deceased sepsis patients, indicating an association between reduced lymphocyte counts and sepsis-related mortality.^[74]^ This conclusion is supported by further research showing that lymphocyte depletion, particularly CD8 + T-cell exhaustion, is a risk factor for sepsis progression.^[75]^ Additionally, studies have indicated that increased CD8+ and CD28 + T-cell counts are negatively correlated with 28-day sepsis-related mortality.^[70]^ The above findings provide theoretical support for our MR results.

In the reverse MR analysis, a significant positive association was confirmed between 28-day sepsis-related mortality and CMV pp28 antibody levels. Transient immune activation occurs in the early stage of sepsis but is soon followed by by immune suppression. Lymphocyte counts decrease persistently by the 4th day after sepsis diagnosis, serving as a biomarker of sepsis-induced immune suppression.^[76]^ Studies have indicated that CMV reactivation commonly manifests in severely immunocompromised septic patients,^[13]^ and its incidence is strongly correlated with immunosuppressive states.^[15,27]^ These findings potentially explain our results, wherein 28-day deceased sepsis patients exhibited elevated CMV pp28 antibody levels. Additionally, we revealed a weak negative association between 28-day sepsis-related mortality and anti-HHV-6 E1A IgG seropositivity. Research has reported that 98.4% of infants and adolescents with sepsis are seropositive for HHV-6.^[11]^ However, deceased sepsis patients experience severe immunosuppression, leading to a reduced immune response to HHV-6, decreased HHV-6 IE1A antibody levels, and ultimately lower anti-HHV-6 E1A IgG levels.

Our MR analysis offers several strengths: first, this study is the 1st to use the largest-scale GWAS database to genetically assess the causal relationships between 46 antibody-mediated immune responses and both sepsis and 28-day sepsis-related mortality. We confirmed that elevated levels of *H pylori* urea antibody and anti-HSV-1 IgG seropositivity are significantly associated with an increased risk of sepsis, whereas elevated CMV pp52 antibody levels are significantly related to a reduced risk of 28-day sepsis-related mortality. Furthermore, 28-day sepsis-related mortality is significantly associated with high levels of CMV pp28 antibody and low anti-HHV-6 E1A IgG seropositivity. Second, additionally, this study is the 1st to perform a 2-step MR analysis using the largest GWAS database to explore the potential mediating role of 91 circulating inflammatory cytokines in the relationship between specific antibody-mediated immune responses and the risks of sepsis and sepsis-related 28-day mortality. We found no evidence that inflammatory cytokines mediate the aforementioned causal relationships. Third, this study utilizes a 2-sample bidirectional MR analysis to investigate the causal relationships between antibody-mediated immune responses and both sepsis and 28-day sepsis-related mortality. We also employed a 2-step MR analysis to explore the mediating effects of 91 circulating inflammatory cytokines, which minimizes common confounding factors and addresses reverse causation (issues often seen in clinical observational studies). Furthermore, the application of various methods (including MR-Egger and MR-PRESSO) in sensitivity analyses ensures the consistency and robustness of our findings. However, the generalizability of our findings is limited to European populations. Differences in genetic architecture, pathogen exposure, and environmental factors across ethnicities may influence the transferability of these causal inferences. Future studies in diverse ethnic populations are therefore essential to confirm their global applicability of our findings.

This study provides insights into future research directions and clinical implications: first, TSMR method was performed to identify causal relationships between genetically predicted levels of *H pylori* urea antibody and anti-HSV-1 IgG seropositivity, and sepsis; as well as between genetically predicted CMV pp52 antibody levels, CMV pp28 antibody levels, anti-HHV-6 E1A IgG seropositivity, and 28-day sepsis-related mortality. However, direct evidence supporting these causal relationships is currently limited. Future studies could conduct randomized controlled trials to directly validate these causal relationships. Large-scale prospective studies could also be conducted to observe the temporal evolution of these relationships over time and their manifestations across different age groups. Second, although our study provides evidence for the aforementioned causal relationships based on genetic predictions, the specific biological and genetic mechanisms underlying these causal relationships remain unclear. Future research should therefore delve into preclinical and clinical studies to further elucidate the biological mechanisms underlying these causal relationships. Third, our mediation analysis provides no evidence that inflammatory cytokines act as mediators of these causal relationships. Future research may shift focus to other potential pathways, such as gut microbiota modulation and T-cell-mediated immunity. Fourth, previous studies have suggested an independent association between EBV seropositivity and increased sepsis-related mortality,^[11]^ but our MR results do not support this causal relationship. Further research is needed to provide additional evidence for this potential causal relationship. Fifth, this study focuses primarily on the European population. Future research should therefore incorporate data from other ethnicities and populations to enhance the generalizability and applicability of the results. Sixth, based on our findings, future interventions targeting *H pylori* urea antibody, anti-HSV-1 IgG, and sepsis could be explored to evaluate the impact of these antibodies on the occurrence and progression of sepsis. In conclusion, our findings provide new insights into the prevention and treatment of sepsis, and contribute to the optimization of sepsis management strategies.

Several limitations of this study warrant consideration. The exclusive use of European GWAS data limits the generalizability of our conclusions. Although the instrumental strength was generally high (*F*-statistics > 10) and sensitivity analyses supported the robustness of our findings, some inter-exposure heterogeneity persisted. Furthermore, while MR reduces confounding, our inferences may be influenced by unmeasured environmental factors and the lack of age or gender stratification in the summary-level GWAS data. Furthermore, the null mediating role of inflammatory cytokines should be interpreted with caution due to limited statistical power, warranting future investigation. Lastly, the observed modest effect sizes and the absence of multiple testing correction underscore the preliminary nature of these findings and highlight the need for independent replication studies.

## 5. Conclusion

Our findings suggest that genetically predicted *H pylori* urea antibody levels and anti-HSV-1 IgG seropositivity are potential pathogenic factors for sepsis, whereas CMV pp52 antibody levels may be protective against 28-day sepsis-related mortality. Reverse MR analysis also indicated potential feedback relationships in severe sepsis cases. Notably, these causal relationships do not appear to be mediated by the common inflammatory cytokines examined in this study. To translate these genetic insights into clinical practice, future large-scale prospective studies are needed to validate the clinical utility of these antibody-mediated immune responses. Subsequent experimental studies should elucidate the underlying mechanisms, such as the roles of gut microbiota modulation and T-cell-mediated cellular immunity, while studies in diverse populations will assess generalizability of our findings. These efforts could pave the way for novel immune-based strategies for sepsis prevention and risk stratification.

## Acknowledgments

We are grateful to the investigators who publicized the GWAS data included in this study. Special thanks to Dr Yun Wen for guiding our project.

## Author contributions

**Conceptualization:** Sheng Xie.

**Methodology:** Lijian Liu.

**Project administration:** Sheng Xie.

**Resources:** Liqun Li.

**Visualization:** Jinjing Tan.

**Writing – original draft:** Liqun Li, Lijian Liu.

**Writing – review & editing:** Jing Yan, Jinjing Tan, Sheng Xie.

## Supplementary Material




