# Supplementary material for: Causal relationships between antibody-induced immune responses and sepsis: Evidence from genetic studies
Source: Medicine (Baltimore). 2026 Feb 13;105(7):e47458. doi: 10.1097/MD.0000000000047458 (PMC12908796; doi:10.1097/MD.0000000000047458)
Supplement: Supplementary file 2 [file medi-105-e47458-s002.pdf]

Trails	method	nsnp	pval	OR(95%CI)
Anti−BK polyomavirus IgG seropositivity	Inverse variance weighted	10	0.7743	0.9966 (0.9737−1.0201)
BK polyomavirus VP1 antibody levels	Inverse variance weighted	10	0.1701	1.0834 (0.9662−1.2147)
Anti−chlamydia trachomatis IgG seropositivity	Inverse variance weighted	9	0.2818	1.0297 (0.9763−1.0860)
Chlamydia trachomatis momp A antibody levels	Inverse variance weighted	17	0.6665	1.0059 (0.9793−1.0332)
Chlamydia trachomatis momp D antibody levels	Inverse variance weighted	6	0.5853	1.0170 (0.9574−1.0803)
Chlamydia trachomatis pGP3 antibody levels	Inverse variance weighted	10	0.1271	1.0416 (0.9885−1.0977)
Chlamydia trachomatis PorB antibody levels	Inverse variance weighted	49	0.6371	0.9975 (0.9873−1.0079)
Chlamydia trachomatis tarp−D F1 antibody levels	Inverse variance weighted	7	0.6184	1.0176 (0.9501−1.0898)
Chlamydia trachomatis tarp−D F2 antibody levels	Inverse variance weighted	12	0.3753	1.0289 (0.9660−1.0959)
Anti−cytomegalovirus IgG seropositivity	Inverse variance weighted	7	0.2150	0.9589 (0.8972−1.0247)
Cytomegalovirus pp28 antibody levels	Inverse variance weighted	12	0.7847	0.9860 (0.8910−1.0911)
Cytomegalovirus pp52 antibody levels	Inverse variance weighted	16	0.1702	0.9421 (0.8652−1.0259)
Cytomegalovirus pp150 antibody levels	Inverse variance weighted	9	0.3085	1.0524 (0.9539−1.1611)
Anti−Epstein−Barr virus IgG seropositivity	Inverse variance weighted	4	0.7043	0.9920 (0.9519−1.0338)
Epstein−Barr virus EA−D antibody levels	Inverse variance weighted	7	0.3865	0.9255 (0.7768−1.1027)
Epstein−Barr virus EBNA−1 antibody levels	Inverse variance weighted	15	0.5773	1.0239 (0.9422−1.1127)
Epstein−Barr virus VCA p18 antibody levels	Inverse variance weighted	24	0.9751	1.0012 (0.9265−1.0820)
Epstein−Barr virus ZEBRA antibody levels	Inverse variance weighted	14	0.0831	0.9438 (0.8841−1.0076)
Anti−human herpes virus 6 IgG seropositivity	Inverse variance weighted	7	0.3736	0.9845 (0.9511−1.0190)
Anti−human herpes virus 6 E1A IgG seropositivity	Inverse variance weighted	14	0.1160	0.9622 (0.9172−1.0095)
Human herpes virus 6 IE1A antibody levels	Inverse variance weighted	13	0.6299	0.9794 (0.9000−1.0658)
Anti−human herpes virus 6 IE1B IgG seropositivity	Inverse variance weighted	9	0.4750	0.9781 (0.9205−1.0393)
Human herpes virus 6 IE1B antibody levels	Inverse variance weighted	16	0.6390	1.0249 (0.9247−1.1360)
Human herpes virus 6 p101k antibody levels	Inverse variance weighted	14	0.3097	0.9712 (0.9181−1.0275)
Anti−human herpes virus 7 IgG seropositivity	Inverse variance weighted	7	0.7164	0.9951 (0.9688−1.0220)
Human herpes virus 7 U14 antibody levels	Inverse variance weighted	13	0.8186	1.0122 (0.9126−1.1227)
Anti−helicobacter pylori IgG seropositivity	Inverse variance weighted	9	0.3005	1.0309 (0.9732−1.0920)
Helicobacter pylori CagA antibody levels	Inverse variance weighted	6	0.8619	0.9954 (0.9448−1.0487)
Helicobacter pylori Catalase antibody levels	Inverse variance weighted	9	0.8813	1.0040 (0.9531−1.0576)
Helicobacter pylori GroEL antibody levels	Inverse variance weighted	4	0.4437	0.9618 (0.8705−1.0626)
Helicobacter pylori OMP antibody levels	Inverse variance weighted	9	0.1378	1.0652 (0.9799−1.1579)
Helicobacter pylori UREA antibody levels	Inverse variance weighted	10	0.0236	1.0696 (1.0091−1.1337)
Helicobacter pylori VacA antibody levels	Inverse variance weighted	16	0.6179	1.0110 (0.9683−1.0556)
Anti−herpes simplex virus 1 IgG seropositivity	Inverse variance weighted	8	0.0184	1.0708 (1.0116−1.1335)
Herpes simplex virus 1 mgG−1 antibody levels	Inverse variance weighted	17	0.6534	0.9771 (0.8831−1.0811)
Anti−herpes simplex virus 2 IgG seropositivity	Inverse variance weighted	8	0.1556	0.9673 (0.9238−1.0127)
Herpes simplex virus 2 mgG−1 antibody levels	Inverse variance weighted	10	0.6562	0.9889 (0.9413−1.0388)
Anti−polyomavirus 2 IgG seropositivity	Inverse variance weighted	10	0.6416	1.0115 (0.9639−1.0615)
Polyomavirus 2 JC VP1 antibody levels	Inverse variance weighted	15	0.8465	1.0073 (0.9361−1.0839)
Anti−Merkel cell polyomavirus IgG seropositivity	Inverse variance weighted	9	0.6370	1.0111 (0.9658−1.0585)
Merkel cell polyomavirus VP1 antibody levels	Inverse variance weighted	11	0.5311	1.0239 (0.9509−1.1026)
Anti−Toxoplasma gondii IgG seropositivity	Inverse variance weighted	12	0.5002	1.0175 (0.9675−1.0701)
Toxoplasma gondii p22 antibody levels	Inverse variance weighted	17	0.4838	1.0140 (0.9752−1.0544)
Toxoplasma gondii sag1 antibody levels	Inverse variance weighted	32	0.7801	1.0062 (0.9637−1.0505)
Anti−varicella zoster virus IgG seropositivity	Inverse variance weighted	11	0.2541	0.9801 (0.9469−1.0145)
Varicella zoster virus glycoproteins E and I antibody levels	Inverse variance weighted	16	0.8018	1.0152 (0.9025−1.1420)

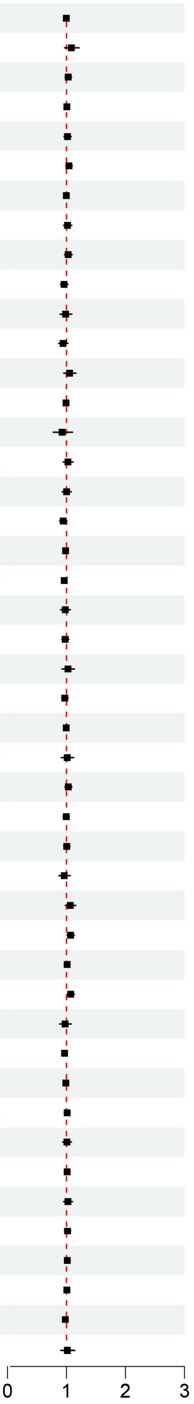

Figure S1:the causal correlations between 46 antibody-mediated immune responses and sepsis (forward MR analysis)

Trails	method	nsnp	pval	OR(95%CI)
Anti−BK polyomavirus IgG seropositivity	Inverse variance weighted	10	0.9880	1.0006 (0.9271−1.0799)
BK polyomavirus VP1 antibody levels	Inverse variance weighted	10	0.2095	1.1957 (0.9045−1.5807)
Anti−chlamydia trachomatis IgG seropositivity	Inverse variance weighted	9	0.8531	1.0105 (0.9046−1.1288)
Chlamydia trachomatis momp A antibody levels	Inverse variance weighted	17	0.8868	0.9946 (0.9235−1.0713)
Chlamydia trachomatis momp D antibody levels	Inverse variance weighted	6	0.9380	1.0087 (0.8114−1.2539)
Chlamydia trachomatis pGP3 antibody levels	Inverse variance weighted	10	0.4971	0.9330 (0.7637−1.1398)
Chlamydia trachomatis PorB antibody levels	Inverse variance weighted	49	0.9492	1.0008 (0.9766−1.0256)
Chlamydia trachomatis tarp−D F1 antibody levels	Inverse variance weighted	7	0.3167	0.9266 (0.7981−1.0757)
Chlamydia trachomatis tarp−D F2 antibody levels	Inverse variance weighted	12	0.4538	1.0501 (0.9241−1.1932)
Anti−cytomegalovirus IgG seropositivity	Inverse variance weighted	7	0.0997	1.1459 (0.9744−1.3475)
Cytomegalovirus pp28 antibody levels	Inverse variance weighted	12	0.2406	1.1245 (0.9244−1.3678)
Cytomegalovirus pp52 antibody levels	Inverse variance weighted	16	0.0484	0.8302 (0.6901−0.9987)
Cytomegalovirus pp150 antibody levels	Inverse variance weighted	9	0.8493	0.9781 (0.7786−1.2287)
Anti−Epstein−Barr virus IgG seropositivity	Inverse variance weighted	4	0.6317	1.0239 (0.9296−1.1279)
Epstein−Barr virus EA−D antibody levels	Inverse variance weighted	7	0.3824	0.8399 (0.5679−1.2423)
Epstein−Barr virus EBNA−1 antibody levels	Inverse variance weighted	15	0.1417	0.8589 (0.7012−1.0521)
Epstein−Barr virus VCA p18 antibody levels	Inverse variance weighted	24	0.8273	0.9818 (0.8324−1.1580)
Epstein−Barr virus ZEBRA antibody levels	Inverse variance weighted	14	0.7845	0.9725 (0.7960−1.1880)
Anti−human herpes virus 6 IgG seropositivity	Inverse variance weighted	7	0.3211	0.9582 (0.8808−1.0425)
Anti−human herpes virus 6 E1A IgG seropositivity	Inverse variance weighted	14	0.0667	0.9147 (0.8315−1.0062)
Human herpes virus 6 IE1A antibody levels	Inverse variance weighted	13	0.8087	1.0288 (0.8178−1.2942)
Anti−human herpes virus 6 IE1B IgG seropositivity	Inverse variance weighted	9	0.7247	1.0208 (0.9104−1.1445)
Human herpes virus 6 IE1B antibody levels	Inverse variance weighted	16	0.9060	1.0119 (0.8314−1.2316)
Human herpes virus 6 p101k antibody levels	Inverse variance weighted	14	0.5749	0.9689 (0.8675−1.0821)
Anti−human herpes virus 7 IgG seropositivity	Inverse variance weighted	7	0.8131	0.9887 (0.8995−1.0867)
Human herpes virus 7 U14 antibody levels	Inverse variance weighted	13	0.2853	0.8824 (0.7015−1.1100)
Anti−helicobacter pylori IgG seropositivity	Inverse variance weighted	9	0.8111	0.9841 (0.8627−1.1225)
Helicobacter pylori CagA antibody levels	Inverse variance weighted	6	0.8414	1.0131 (0.8919−1.1507)
Helicobacter pylori Catalase antibody levels	Inverse variance weighted	9	0.9676	1.0026 (0.8831−1.1384)
Helicobacter pylori GroEL antibody levels	Inverse variance weighted	4	0.5661	0.9055 (0.6452−1.2709)
Helicobacter pylori OMP antibody levels	Inverse variance weighted	9	0.8058	1.0240 (0.8475−1.2373)
Helicobacter pylori UREA antibody levels	Inverse variance weighted	10	0.2561	1.0860 (0.9419−1.2522)
Helicobacter pylori VacA antibody levels	Inverse variance weighted	16	0.3348	1.0462 (0.9544−1.1468)
Anti−herpes simplex virus 1 IgG seropositivity	Inverse variance weighted	8	0.2727	1.0808 (0.9407−1.2419)
Herpes simplex virus 1 mgG−1 antibody levels	Inverse variance weighted	17	0.2353	0.8907 (0.7357−1.0783)
Anti−herpes simplex virus 2 IgG seropositivity	Inverse variance weighted	8	0.5710	0.9639 (0.8489−1.0945)
Herpes simplex virus 2 mgG−1 antibody levels	Inverse variance weighted	10	0.9612	1.0027 (0.8977−1.1200)
Anti−polyomavirus 2 IgG seropositivity	Inverse variance weighted	10	0.8545	1.0096 (0.9120−1.1176)
Polyomavirus 2 JC VP1 antibody levels	Inverse variance weighted	15	0.5420	0.9526 (0.8148−1.1136)
Anti−Merkel cell polyomavirus IgG seropositivity	Inverse variance weighted	9	0.5924	0.9699 (0.8672−1.0848)
Merkel cell polyomavirus VP1 antibody levels	Inverse variance weighted	11	0.6387	0.9596 (0.8076−1.1400)
Anti−Toxoplasma gondii IgG seropositivity	Inverse variance weighted	12	0.5487	0.9610 (0.8438−1.0945)
Toxoplasma gondii p22 antibody levels	Inverse variance weighted	17	0.6951	1.0201 (0.9234−1.1270)
Toxoplasma gondii sag1 antibody levels	Inverse variance weighted	32	0.1158	1.0848 (0.9802−1.2007)
Anti−varicella zoster virus IgG seropositivity	Inverse variance weighted	11	0.3217	0.9584 (0.8811−1.0424)
Varicella zoster virus glycoproteins E and I antibody levels	Inverse variance weighted	16	0.4396	1.0742 (0.8959−1.2879)

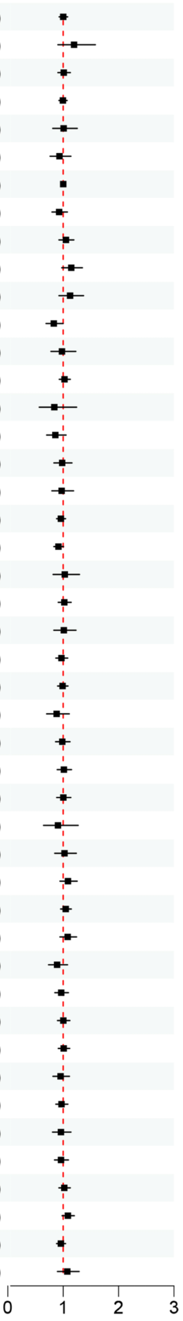

Figure S2:the causal correlations between 46 antibody-mediated immune responses and 28day death of sepsis (forward MR analysis)

Trails	method	nsnp	pval	OR(95%CI)
Anti−BK polyomavirus IgG seropositivity	Inverse variance weighted	18	0.3884	1.2646 (0.7418−2.1556)
BK polyomavirus VP1 antibody levels	Inverse variance weighted	18	0.1840	1.0802 (0.9640−1.2104)
Anti−chlamydia trachomatis IgG seropositivity	Inverse variance weighted	18	0.7267	1.0607 (0.7621−1.4764)
Chlamydia trachomatis momp A antibody levels	Inverse variance weighted	18	0.8394	0.9670 (0.6986−1.3383)
Chlamydia trachomatis momp D antibody levels	Inverse variance weighted	18	0.6999	1.0586 (0.7924−1.4144)
Chlamydia trachomatis pGP3 antibody levels	Inverse variance weighted	18	0.3851	1.1311 (0.8566−1.4937)
Chlamydia trachomatis PorB antibody levels	Inverse variance weighted	17	0.3665	0.7456 (0.3942−1.4102)
Chlamydia trachomatis tarp−D F1 antibody levels	Inverse variance weighted	18	0.4440	0.8985 (0.6832−1.1817)
Chlamydia trachomatis tarp−D F2 antibody levels	Inverse variance weighted	18	0.9475	1.0076 (0.8042−1.2625)
Anti−cytomegalovirus IgG seropositivity	Inverse variance weighted	18	0.9409	0.9913 (0.7869−1.2488)
Cytomegalovirus pp28 antibody levels	Inverse variance weighted	18	0.8239	1.0234 (0.8348−1.2547)
Cytomegalovirus pp52 antibody levels	Inverse variance weighted	18	0.6306	1.0473 (0.8674−1.2646)
Cytomegalovirus pp150 antibody levels	Inverse variance weighted	18	0.3283	0.9273 (0.7971−1.0788)
Anti−Epstein−Barr virus IgG seropositivity	Inverse variance weighted	18	0.7258	0.9151 (0.5575−1.5022)
Epstein−Barr virus EA−D antibody levels	Inverse variance weighted	18	0.6881	0.9745 (0.8590−1.1055)
Epstein−Barr virus EBNA−1 antibody levels	Inverse variance weighted	18	0.5835	1.0348 (0.9158−1.1692)
Epstein−Barr virus VCA p18 antibody levels	Inverse variance weighted	18	0.1229	1.0981 (0.9750−1.2368)
Epstein−Barr virus ZEBRA antibody levels	Inverse variance weighted	18	0.8730	0.9896 (0.8709−1.1245)
Anti−human herpes virus 6 IgG seropositivity	Inverse variance weighted	18	0.3572	1.2226 (0.7970−1.8756)
Anti−human herpes virus 6 E1A IgG seropositivity	Inverse variance weighted	18	0.2718	1.1955 (0.8694−1.6440)
Human herpes virus 6 IE1A antibody levels	Inverse variance weighted	18	0.9006	0.9920 (0.8742−1.1256)
Anti−human herpes virus 6 IE1B IgG seropositivity	Inverse variance weighted	18	0.1204	1.2477 (0.9437−1.6497)
Human herpes virus 6 IE1B antibody levels	Inverse variance weighted	18	0.9582	1.0034 (0.8854−1.1371)
Human herpes virus 6 p101k antibody levels	Inverse variance weighted	18	0.7788	1.0345 (0.8164−1.3109)
Anti−human herpes virus 7 IgG seropositivity	Inverse variance weighted	18	0.3173	1.4618 (0.6945−3.0768)
Human herpes virus 7 U14 antibody levels	Inverse variance weighted	18	0.8030	0.9830 (0.8589−1.1250)
Anti−helicobacter pylori IgG seropositivity	Inverse variance weighted	18	0.2912	1.1445 (0.8908−1.4706)
Helicobacter pylori CagA antibody levels	Inverse variance weighted	18	0.5294	0.9003 (0.6490−1.2488)
Helicobacter pylori Catalase antibody levels	Inverse variance weighted	18	0.8891	1.0191 (0.7812−1.3295)
Helicobacter pylori GroEL antibody levels	Inverse variance weighted	18	0.6500	1.0542 (0.8393−1.3241)
Helicobacter pylori OMP antibody levels	Inverse variance weighted	18	0.5931	0.9428 (0.7597−1.1701)
Helicobacter pylori UREA antibody levels	Inverse variance weighted	18	0.9054	1.0163 (0.7788−1.3261)
Helicobacter pylori VacA antibody levels	Inverse variance weighted	18	0.8797	1.0201 (0.7881−1.3204)
Anti−herpes simplex virus 1 IgG seropositivity	Inverse variance weighted	18	0.8550	0.9733 (0.7283−1.3008)
Herpes simplex virus 1 mgG−1 antibody levels	Inverse variance weighted	18	0.2918	1.0836 (0.9334−1.2580)
Anti−herpes simplex virus 2 IgG seropositivity	Inverse variance weighted	18	0.1381	0.7901 (0.5787−1.0788)
Herpes simplex virus 2 mgG−1 antibody levels	Inverse variance weighted	18	0.2253	1.1889 (0.8988−1.5726)
Anti−polyomavirus 2 IgG seropositivity	Inverse variance weighted	18	0.0628	0.8052 (0.6409−1.0117)
Polyomavirus 2 JC VP1 antibody levels	Inverse variance weighted	18	0.7037	1.0289 (0.8882−1.1919)
Anti−Merkel cell polyomavirus IgG seropositivity	Inverse variance weighted	18	0.0802	0.8084 (0.6370−1.0259)
Merkel cell polyomavirus VP1 antibody levels	Inverse variance weighted	18	0.4454	1.0569 (0.9169−1.2182)
Anti−Toxoplasma gondii IgG seropositivity	Inverse variance weighted	18	0.9430	1.0093 (0.7833−1.3005)
Toxoplasma gondii p22 antibody levels	Inverse variance weighted	18	0.5926	1.1022 (0.7717−1.5744)
Toxoplasma gondii sag1 antibody levels	Inverse variance weighted	18	0.9634	0.9959 (0.8347−1.1881)
Anti−varicella zoster virus IgG seropositivity	Inverse variance weighted	18	0.5566	0.9104 (0.6657−1.2450)
Varicella zoster virus glycoproteins E and I antibody levels	Inverse variance weighted	18	0.5243	1.0400 (0.9218−1.1733)

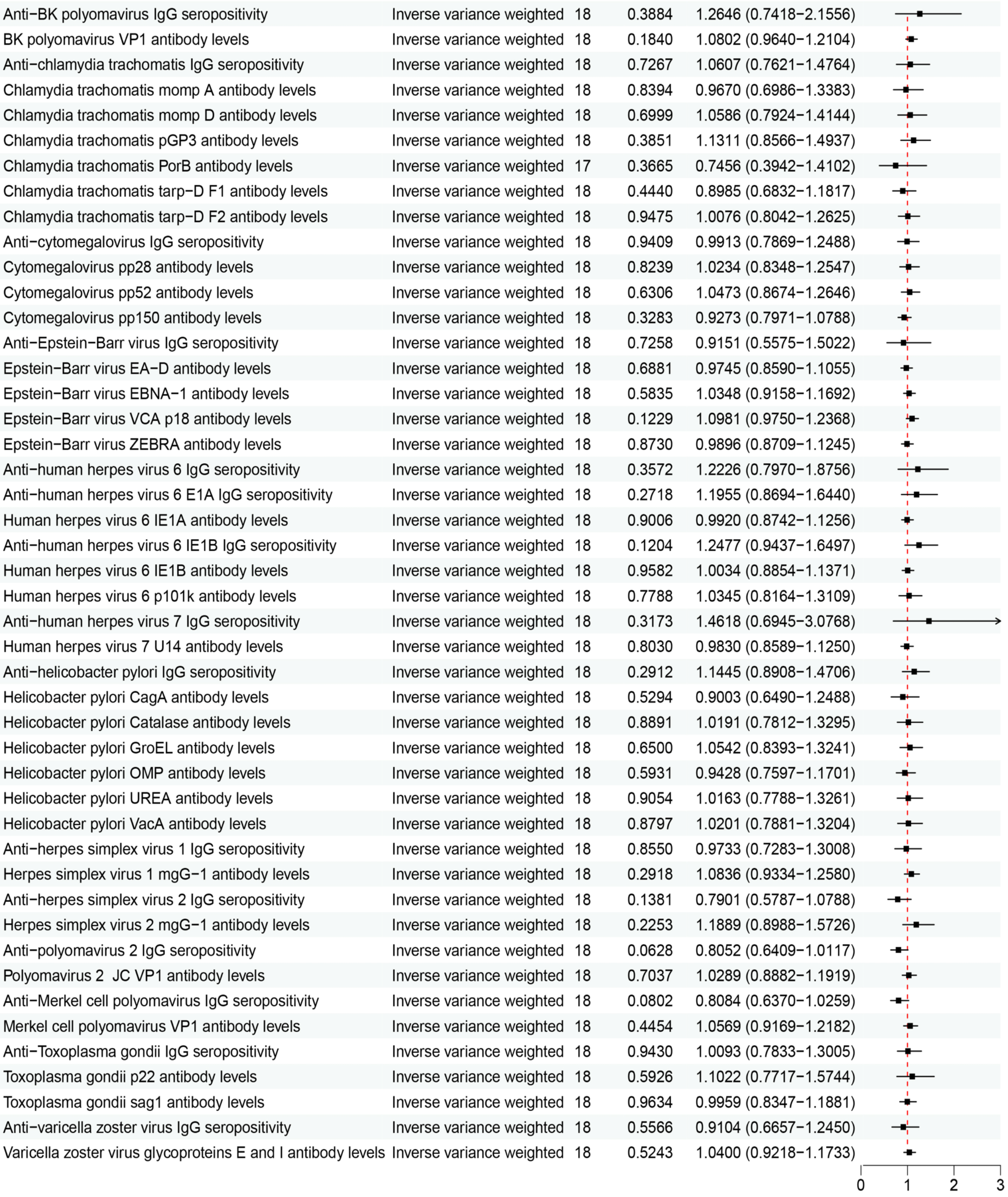

Figure S3:the causal correlations between 46 antibody-mediated immune responses and sepsis (reverse MR analysis)

Trails	method	nsnp	pval	OR(95%CI)
Anti−BK polyomavirus IgG seropositivity	Inverse variance weighted	24	0.3762	1.0905 (0.9001−1.3210)
BK polyomavirus VP1 antibody levels	Inverse variance weighted	24	0.7775	1.0059 (0.9657−1.0478)
Anti−chlamydia trachomatis IgG seropositivity	Inverse variance weighted	24	0.7093	0.9771 (0.8653−1.1034)
Chlamydia trachomatis momp A antibody levels	Inverse variance weighted	24	0.9070	1.0077 (0.8854−1.1470)
Chlamydia trachomatis momp D antibody levels	Inverse variance weighted	24	0.9672	0.9975 (0.8847−1.1247)
Chlamydia trachomatis pGP3 antibody levels	Inverse variance weighted	24	0.9803	1.0012 (0.9112−1.1000)
Chlamydia trachomatis PorB antibody levels	Inverse variance weighted	24	0.1279	0.8513 (0.6920−1.0473)
Chlamydia trachomatis tarp−D F1 antibody levels	Inverse variance weighted	24	0.2906	1.0507 (0.9586−1.1515)
Chlamydia trachomatis tarp−D F2 antibody levels	Inverse variance weighted	24	0.1843	1.0576 (0.9737−1.1487)
Anti−cytomegalovirus IgG seropositivity	Inverse variance weighted	24	0.4764	1.0305 (0.9486−1.1195)
Cytomegalovirus pp28 antibody levels	Inverse variance weighted	24	<0.05	1.0555 (1.0011−1.1128)
Cytomegalovirus pp52 antibody levels	Inverse variance weighted	24	0.3737	1.0227 (0.9733−1.0746)
Cytomegalovirus pp150 antibody levels	Inverse variance weighted	24	0.8452	1.0053 (0.9530−1.0606)
Anti−Epstein−Barr virus IgG seropositivity	Inverse variance weighted	24	0.3125	1.0984 (0.9155−1.3177)
Epstein−Barr virus EA−D antibody levels	Inverse variance weighted	24	0.8454	1.0043 (0.9622−1.0482)
Epstein−Barr virus EBNA−1 antibody levels	Inverse variance weighted	24	0.8560	1.0040 (0.9612−1.0488)
Epstein−Barr virus VCA p18 antibody levels	Inverse variance weighted	24	0.8480	1.0040 (0.9635−1.0463)
Epstein−Barr virus ZEBRA antibody levels	Inverse variance weighted	24	0.5807	1.0118 (0.9706−1.0547)
Anti−human herpes virus 6 IgG seropositivity	Inverse variance weighted	24	0.3461	0.9338 (0.8099−1.0768)
Anti−human herpes virus 6 E1A IgG seropositivity	Inverse variance weighted	24	<0.05	0.8949 (0.8114−0.9870)
Human herpes virus 6 IE1A antibody levels	Inverse variance weighted	24	0.7860	1.0072 (0.9563−1.0608)
Anti−human herpes virus 6 IE1B IgG seropositivity	Inverse variance weighted	24	0.7929	1.0135 (0.9169−1.1203)
Human herpes virus 6 IE1B antibody levels	Inverse variance weighted	24	0.3888	0.9804 (0.9373−1.0255)
Human herpes virus 6 p101k antibody levels	Inverse variance weighted	24	0.3541	0.9564 (0.8703−1.0510)
Anti−human herpes virus 7 IgG seropositivity	Inverse variance weighted	24	0.0929	1.1964 (0.9706−1.4748)
Human herpes virus 7 U14 antibody levels	Inverse variance weighted	24	0.2363	0.9757 (0.9369−1.0162)
Anti−helicobacter pylori IgG seropositivity	Inverse variance weighted	24	0.4187	1.0378 (0.9485−1.1355)
Helicobacter pylori CagA antibody levels	Inverse variance weighted	24	0.6550	1.0282 (0.9101−1.1616)
Helicobacter pylori Catalase antibody levels	Inverse variance weighted	24	0.5418	1.0312 (0.9343−1.1381)
Helicobacter pylori GroEL antibody levels	Inverse variance weighted	24	0.9687	0.9986 (0.9289−1.0734)
Helicobacter pylori OMP antibody levels	Inverse variance weighted	24	0.6815	0.9846 (0.9145−1.0602)
Helicobacter pylori UREA antibody levels	Inverse variance weighted	24	0.7858	1.0109 (0.9347−1.0935)
Helicobacter pylori VacA antibody levels	Inverse variance weighted	24	0.8129	1.0114 (0.9206−1.1113)
Anti−herpes simplex virus 1 IgG seropositivity	Inverse variance weighted	24	0.4064	0.9605 (0.8733−1.0564)
Herpes simplex virus 1 mgG−1 antibody levels	Inverse variance weighted	24	0.5364	0.9850 (0.9388−1.0334)
Anti−herpes simplex virus 2 IgG seropositivity	Inverse variance weighted	24	0.5991	0.9705 (0.8678−1.0853)
Herpes simplex virus 2 mgG−1 antibody levels	Inverse variance weighted	24	0.9593	1.0026 (0.9085−1.1064)
Anti−polyomavirus 2 IgG seropositivity	Inverse variance weighted	24	0.3832	1.0382 (0.9543−1.1294)
Polyomavirus 2 JC VP1 antibody levels	Inverse variance weighted	24	0.8922	1.0040 (0.9471−1.0644)
Anti−Merkel cell polyomavirus IgG seropositivity	Inverse variance weighted	24	0.4686	1.0321 (0.9475−1.1242)
Merkel cell polyomavirus VP1 antibody levels	Inverse variance weighted	24	0.5561	0.9852 (0.9375−1.0353)
Anti−Toxoplasma gondii IgG seropositivity	Inverse variance weighted	24	0.4630	1.0346 (0.9447−1.1332)
Toxoplasma gondii p22 antibody levels	Inverse variance weighted	24	0.4750	1.0382 (0.9367−1.1507)
Toxoplasma gondii sag1 antibody levels	Inverse variance weighted	24	0.1595	1.0449 (0.9829−1.1108)
Anti−varicella zoster virus IgG seropositivity	Inverse variance weighted	24	0.6013	1.0369 (0.9050−1.1881)
Varicella zoster virus glycoproteins E and I antibody levels	Inverse variance weighted	24	0.9603	1.0011 (0.9587−1.0453)

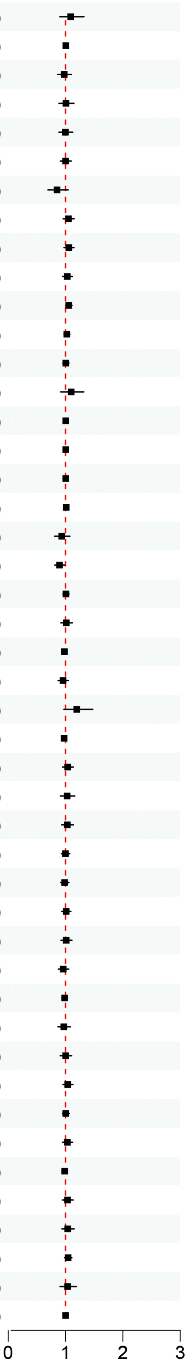

Figure S4 the causal correlations between 46 antibody-mediated immune responses and 28-day death of sepsis (reverse MR analysis)
